# Preclinical efficacy of maternal embryonic leucine-zipper kinase (MELK) inhibition in acute myeloid leukemia

**DOI:** 10.18632/oncotarget.2642

**Published:** 2014-10-28

**Authors:** Houda Alachkar, Martin B.G. Mutonga, Klaus H. Metzeler, Noreen Fulton, Gregory Malnassy, Tobias Herold, Karsten Spiekermann, Stefan K. Bohlander, Wolfgang Hiddemann, Yo Matsuo, Wendy Stock, Yusuke Nakamura

**Affiliations:** ^1^ Department of Medicine, University of Chicago, Chicago, IL; ^2^ Department of Internal Medicine 3, University Hospital Grosshadern, Ludwig-Maximilians-Universität (LMU), München, Germany; ^3^ Clinical Cooperative Group Leukemia, Helmholtz Center Munich for Environmental Health, München, Germany; ^4^ Department of Molecular Medicine and Pathology, The University of Auckland, Auckland, New Zealand; ^5^ OncoTherapy Science, Inc., Kanagawa, Japan

**Keywords:** MELK, AML, OTS167

## Abstract

Maternal embryonic leucine-zipper kinase (MELK), which was reported to be frequently up-regulated in various types of solid cancer, plays critical roles in formation and maintenance of cancer stem cells. However, little is known about the relevance of this kinase in hematologic malignancies. Here we report characterization of possible roles of MELK in acute myeloid leukemia (AML). MELK is expressed in AML cell lines and AML blasts with higher levels in less differentiated cells. MELK is frequently upregulated in AML with complex karyotypes and is associated with worse clinical outcome. MELK knockdown resulted in growth inhibition and apoptosis of leukemic cells. Hence, we investigated the potent anti-leukemia activity of OTS167, a small molecule MELK kinase inhibitor, in AML, and found that the compound induced cell differentiation and apoptosis as well as decreased migration of AML cells. MELK expression was positively correlated with the expression of FOXM1 as well as its downstream target genes. Furthermore, MELK inhibition resulted in downregulation of FOXM1 activity and the expression of its downstream targets. Taken together, and given that OTS167 is undergoing a phase I clinical trial in solid cancer, our study warrants clinical evaluation of this compound as a novel targeted therapy for AML patients.

## INTRODUCTION

MELK (maternal embryonic leucine zipper kinase) also known as MPK38 is a cell-cycle dependent protein kinase that belongs to the AMP-activated Ser/Thr protein kinase family [1, 2]. In normal adult tissues, MELK mRNA expression was hardly detectable except in testis and at very low levels in the thymus and small intestine [3, 4]. In addition, MELK was reported to be expressed in neural progenitors and hematopoietic stem cells [5].

MELK was found to be up-regulated in various types of cancer including breast cancer [3] and glioblastoma [6, 7]. Moreover, high levels of *MELK* expression correlated with poorly differentiated histological types of brain tumor and prostate cancer [8-10], and is associated with poor prognosis of patients with breast cancer [11]. Several studies have shown that down-regulation of MELK by treatment with siRNA significantly induced apoptosis in breast cancer cells and various types of brain tumor [3, 6]. Additionally, *MELK* was identified as one of the genes commonly expressed in undifferentiated cancer cells which may suggest a possible role for MELK in cancer stem cell maintenance and survival [12].

MELK also contributes to cell cycle progression and proliferation, likely through phosphorylation of CDC25b [7, 13]. MELK was found to be activated by autophosphorylation [14], however, it is not clear what triggers this self-activation and whether a specific substrate binding is required for autophosphorylation. Several substrates for MELK have been reported; for example, in glioblastoma stem cells, MELK was found to phosphorylate FOXM1, a crucial transcription factor and a master regulator of mitosis in cancer stem cells [15]. FOXM1 and its targets such as Cyclin B1 have been implicated in promoting proliferation through modulating cell cycle progression in acute myeloid leukemia (AML) [16]. Therefore, it is plausible that targeting MELK in AML may affect cell proliferation and cell cycle progression, and thus may provide a therapeutic advantage. MELK was reported to be expressed in hematopoietic cells [4], and likely to be involved in hematopoiesis as demonstrated in a zebra fish model [17]. However, the expression and the function of MELK in hematological malignancies have not yet been characterized. AML is a clonal disease derived from the hematopoietic stem cells; therefore similar to glioblastoma stem cells, we speculated that MELK-dependent mechanisms might play an important role in leukemia stem-cell survival and proliferation. Here we aimed to characterize the expression of MELK in AML and examine possible biological roles of this gene in the pathogenesis of this disease. We demonstrate that MELK is expressed in AML cell lines and in AML primary blasts and that the expression of this gene is significantly higher in the stem cell-enriched population of blast cells obtained from AML patients than that in the more differentiated cell population. Targeting MELK expression with siRNA or MELK kinase activity with a small molecule inhibitor (OTS167) [18, 19] resulted in significant growth inhibition of AML cells. Furthermore, we demonstrate the effect of MELK inhibition on FOXM1 and its downstream targets. Importantly, OTS167 induced myeloid differentiation and apoptosis and also decreased cell migration. Our study suggests that MELK is potentially an important novel therapeutic target in AML and clinical development of OTS167 in AML warrants consideration.

## RESULTS

### MELK expression in AML patients and association with clinical outcome

**Figure 1:**
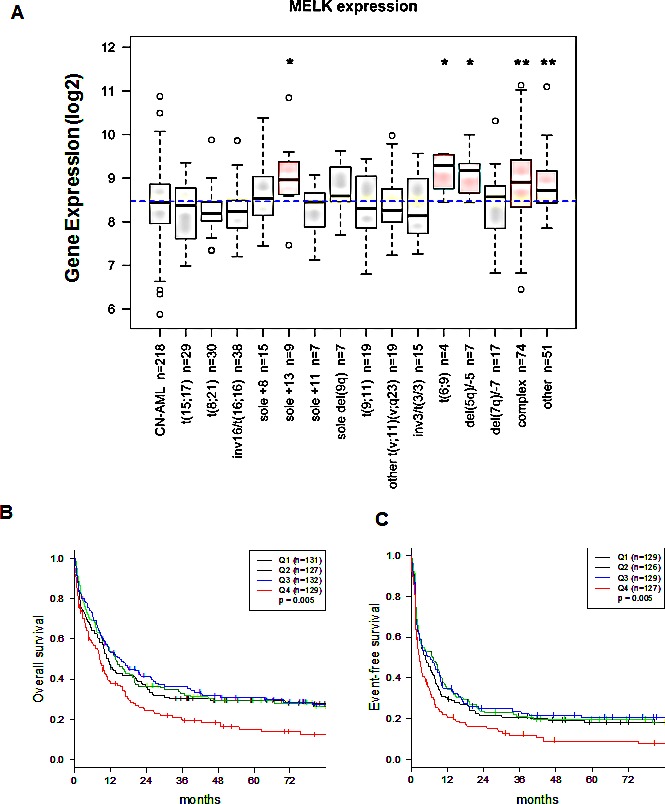
*MELK* mRNA expression levels in blasts of AML patients are correlated with clinical outcome (A) *MELK* mRNA expression levels in blasts of AML patients according to patient's cytogenetic abnormalities. The X axis represents the different cytogenetic subsets of AML and the Y axis represents *MELK* mRNA expression. Boxplot of *MELK* expression in 559 adult AML patients; the blue dashed line indicates the median *MELK* expression in all samples. The Kruskal-Wallis P value for overall heterogeneity across all subgroups was <0.0001. Wilcoxon test was used to compare *MELK* expression of each cytogenetically abnormal subgroup to patients with cytogenetically normal (CN-) AML (reference). An asterisk (*) denotes Wilcoxon's P < 0.05 and a double asterisk (**) denotes P < 0.0001. Patients were divided into 4 groups based on quartiles of MELK expression (Q1: lowest 25%, Q2: 25^th^ percentile - median, Q3: median – 75^th^ percentile, Q4: highest 25%) (B) Association between MELK expression and overall survival (OS). (C) Association between MELK expression and event free survival (EFS).

*MELK* expression was assessed by gene expression microarray in primary AML cells from 559 (age range, 18-59 years) adult patients at the time of diagnosis. Clinical and molecular characteristics of these patients have been previously reported [20]. We found MELK to be expressed at variable levels in different subsets of AML patients. Interestingly, AML patients with complex karyotype (Wilcoxon's P <0.0001), t(6,9) and del(5q)/−5 (Wilcoxon's P < 0.05), were found to have relatively higher levels of MELK expression than other subsets (Figure 1A). Survival analyses were performed in a cohort (n=519 patients) restricted to patients with available data on survival and cytogenetics, and patients with t(15;17) (i.e. APL patients) were excluded due to different biology and treatment. Considering *MELK* expression as a continuous variable, patients expressing higher levels of *MELK* transcript revealed significantly shorter event-free survival (EFS; 3.8 vs 6.5 months; P = 0.02) and shorter overall survival (OS; 11.2 vs 12.9; P = 0.04). When patients were classified according to quartiles of MELK expression, those in the highest quartile had shorter OS and EFS (Log-rank test, P=0.005, both; for the comparison across all 4 groups) (Figure 1B and C) and lower complete remission rate (Fisher exact test, P = 0.03).

### MELK expression in AML cell lines and primary blast cells

**Figure 2:**
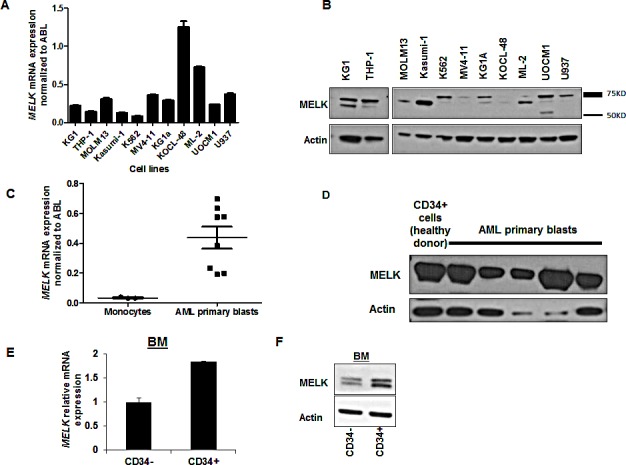
MELK expression in AML cell lines and primary blast cells (A) *MELK* mRNA and (B) protein expression in 11 AML cell lines. (C) *MELK* mRNA and (D) protein expression in primary blasts from AML patients. (E) *MELK* mRNA and (F) protein expression in sorted CD34-positive and -negative cells isolated from bone marrow blasts (BM) of an AML patient. Error bars represent standard error (SE).

We assessed *MELK* mRNA expression by quantitative real-time PCR (qRT-PCR) in 11 AML cell lines representative of the different cytogenetic and molecular subsets of the disease. MELK transcript was expressed at variable levels in all AML cell lines (Figure 2A). MELK protein was also detected in all AML cell lines but was not well correlated with its transcript level, suggesting the presence of some mechanisms that may regulate its translation and the protein stability. Importantly, we observed several isoforms of MELK protein expressed at different levels among the different AML cell lines (Figure 2B). Furthermore, we examined *MELK* expression in primary blasts obtained from eight patients with AML by qRT-PCR, and compared it with that in monocytes obtained from three healthy donors; we found *MELK* expression to be significantly higher in AML blasts compared to that in monocytes (P = 0.01; Figure 2C). Additionally, we measured MELK protein level in nine AML patients by western blot analysis, and found MELK to be expressed in all the samples examined. MELK protein was also detected in normal mobilized CD34+ cells obtained from a healthy donor (Figure 2D, shows five representative patient samples).

Interestingly, MELK transcript and protein were observed at higher levels in CD34+ cells than CD34- cells isolated from AML blasts, measured by qRT-PCR and western blot analyses, respectively (Figure 2E,F).

### MELK knock-down decreased cell viability and induced apoptosis in AML cell lines

**Figure 3:**
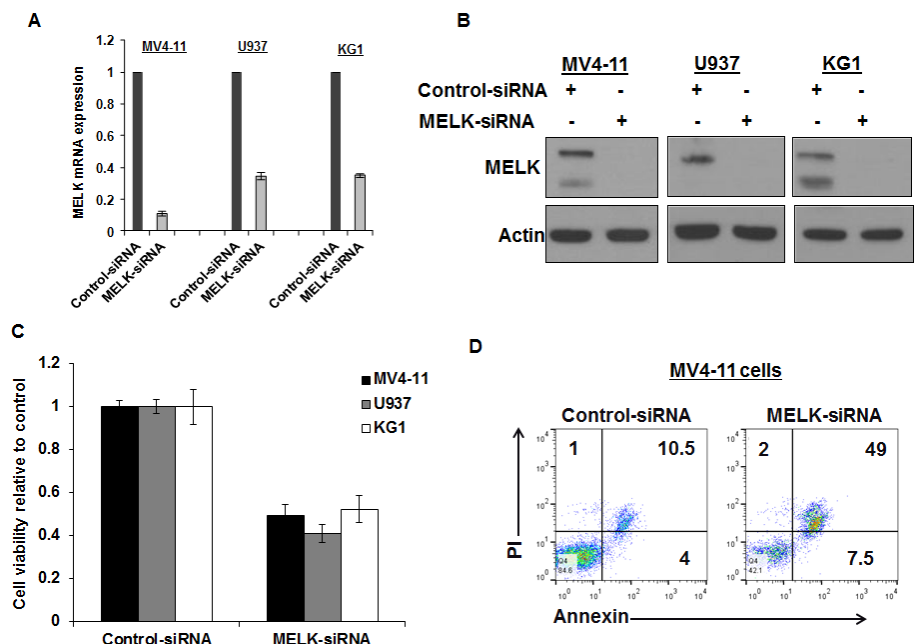
MELK knock-down decreased cell viability and induced apoptosis in AML cell lines (A) *MELK* mRNA expression, (B) protein expression and (C) cell viability in MV4-11, U937 and KG1 cells transfected with *MELK*-siRNA relative to cells transfected with control-siRNA. (D) Annexin and PI staining in MV4-11 cells transfected with *MELK*-siRNA or control siRNA (the numbers represent the percentage of cells in each quarter).

In order to assess the biological function of MELK in AML, we applied a loss of function approach using three AML cell lines (MV4-11, U937 and KG1) that expressed variable levels of MELK. We confirmed *MELK* knockdown by qRT-PCR (Figure 3A) and western blot 48 hours post-transfection and (Figure 3B) found that all three cell lines transfected with *MELK*-siRNA showed significant decrease in cell viability compared with those transfected with control-siRNA (P < 0.05 for all of the three cell lines) (Figure 3C). These results were also validated in MV4-11 cells using two other siRNAs against *MELK* (Figure S1). Furthermore, cells transfected with *MELK*-siRNA exhibited significant increase in apoptosis (~35%, P = 0.03) when compared with cells transfected with control-siRNA by the measurement of Annexin and PI staining (Figure 3D).

### MELK inhibitor OTS167 exhibits anti-leukemia activity in AML cell lines

**Figure 4:**
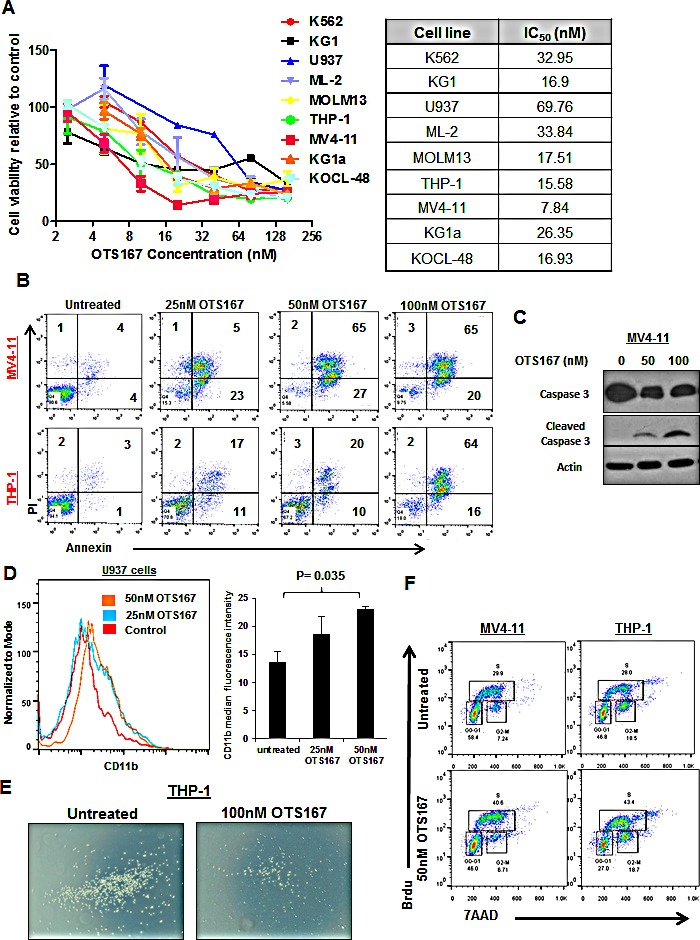
MELK inhibitor OTS167 exhibits anti-leukemia activity in AML cell lines (A) MTT assays for nine AML cell lines after 48-hour incubation with OTS167 at indicated doses. (B) Apoptosis assessed by Annexin and PI staining in MV4-11 and THP-1 cells treated with 25, 50 or 100nM of OTS167 for 72 hours (the numbers represent the percentage of cells in each quarter). (C) Western blot showing cleaved caspase-3 in MV4-11 cells treated with 50 and 100nM OTS167 for 48 hours. (D) Myeloid differentiation assessed by CD11b staining in U937 cells treated with 25 or 50nM of OTS167 and quantification of CD11b staining. (E) Migration of THP-1 cells treated with 100nM of OTS167 for 6 hours. (F) Cell cycle analysis performed in MV4-11 and THP-1 cells treated with 50nM of OTS167 for 24 hours, and assayed using 7-aminoactinomycin D (7AAD) and Bromodeoxyuridin (Brdu) staining.

We utilized a recently developed and very potent MELK inhibitor OTS167 (IC_50_ = 0.41 nM) [18] and treated nine different AML cell lines at various doses of OTS167. Cells showed variable sensitivities to the compound with IC_50_ ranging from 8nM to 70nM (Figure 4A). MV4-11 cells treated with 25nM, 50nM or 100nM of OTS167 revealed 30-40% increase in the apoptotic population at 48 hours and 70-80% (P ≤ 0.001) at 72 hours. THP-1 cells showed 7-40% increase in apoptosis at 48 hours and 20-75% (P ≤ 0.01) at 72 hours post treatment with OTS167 (Figure 4B). OTS167 at 50 and 100nM also induced activation of Caspase 3 indicated by the induction of cleaved Caspase-3 in MV4-11 cells, further validating the apoptosis-inducible activity of this compound (Figure 4C). Importantly, increase in myeloid differentiation was observed in U937 cells treated with 50nM of OTS167 compared with untreated cells when assessed by CD11b staining (P = 0.04; Figure 4D). In addition, pretreatment with 100nM of OTS167 for 6 hours inhibited the migration of THP-1 cells towards the chemoattractant agent SDF-1, as evident by the decrease in the number of migrated cells (Figure 4E and S2). No significant increase in apoptosis was observed in these cells at 12 hours following treatment, suggesting that the effect on migration is not due to the increase in cell death (Figure S3).

Additionally, cell cycle analysis performed in MV4-11 and THP-1 cells showed that treatment with 50nM of OTS167 resulted in significant decrease in the percentage of cells in G0-G1 phase (OTS167 vs untreated; 47% vs 55%; P = 0.02, and 28% vs 43% P = 0.006 in MV4-11 and THP-1 cells, respectively). We also observed an increase in the percentage of cells in the S phase (OTS167 vs untreated; 38% vs 31%; P = 0.05, and 42% vs 28% P = 0.002 in MV4-11 and THP-1 cells, respectively; Figure 4F shows a representative experiment out of three performed) suggesting possible inhibition in the process of the S to G2-M transition; under these conditions, apoptosis was assessed to confirm that this effect was not due to increase in apoptosis (Figure S4).

### Growth suppressive effect of MELK inhibitor OTS167 on MLL-AF9 mouse cells and AML primary blasts

**Figure 5:**
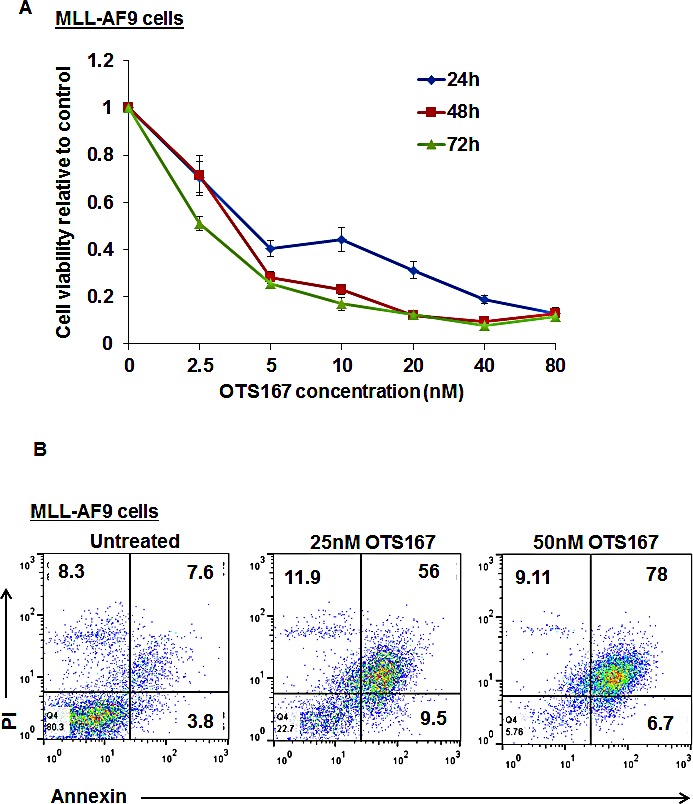
Growth suppressive effect of MELK inhibitor OTS167 on MLL-AF9 mouse cells (A) MTT assay in MLL-AF9 cell line was performed after 48-hour incubation with OTS167 at indicated doses. (B) Apoptosis assessed by Annexin and PI staining in MLL-AF9 cells treated with 25 or 50nM of OTS167 for 48 hours, (the numbers represent the percentage of cells in each quarter).

To confirm the growth-inhibitory activity of OTS167 in AML, we used a cell line in which the human MLL-AF9 oncogene was introduced into mouse hematopoietic cells. The viability of MLL-AF9 cells was dramatically affected by OTS167 in a time- and dose-dependent manner as shown in Figure 5A. In addition, the exposure of this cell line to 25 and 50nM of OTS167 resulted in a 57% and 74% increase in apoptotic cell death, respectively, assessed by Annexin and PI staining (P = 0.001 for each; Figure 5B).

**Figure 6:**
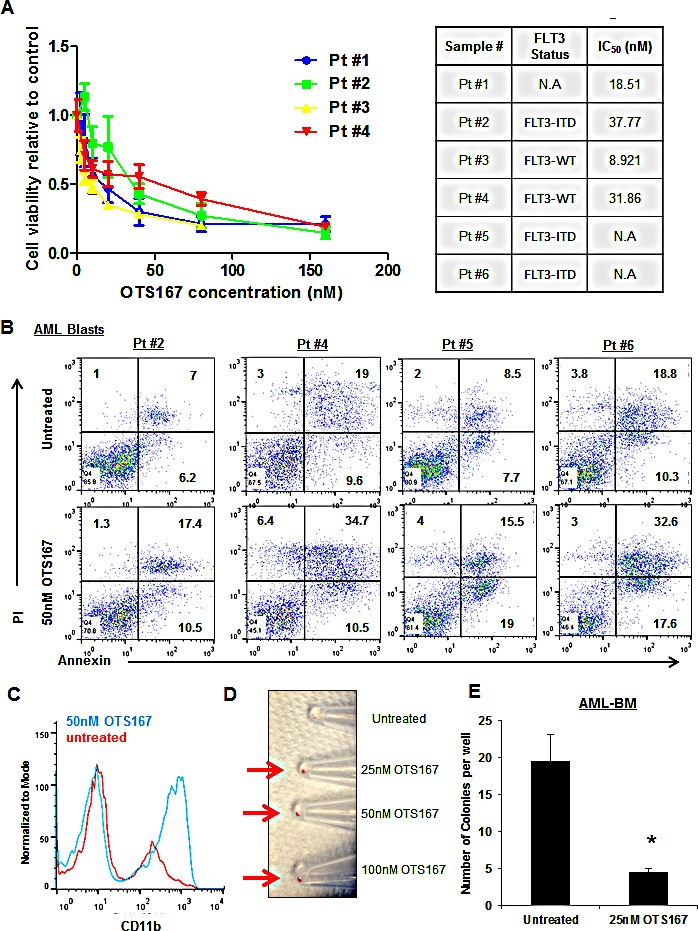
Growth suppressive effect of MELK inhibitor OTS167 in AML primary blasts (A) MTT assay performed in blasts from four AML patients after 48-hour incubation with OTS167 at indicated doses. (B) Apoptosis assessed by Annexin and PI staining in primary blasts treated with 50nM of OTS167 for 72 hours (the numbers represent the percentage of cells in each quarter). (C) Myeloid differentiation assessed by CD11b staining in primary blasts treated with 50nM of OTS167 for 4 days. (D) Cell pellets of primary blasts treated with 25, 50, or 100nM of OTS167 for 4 days (an arrow points to the cell pellet). (E) Colony assay were performed in bone marrow blasts (BM) treated with 25nM OTS167 for 24 hours before plating; error bars represent standard error (SE).

To further validate our findings, we treated blasts obtained from four different patients with AML with various doses of OTS167 (Figure 6A) and found the IC_50_ values for these cells were 18.5, 37.7, 8.9, and 31.8nM. In addition, we assessed apoptosis by flow cytometry of blasts obtained from four patients with AML and observed ~20% increase in apoptosis when cells were treated with 50nM of OTS167 (P = 0.04; Figure 6B). Furthermore, we observed potential differentiation of primary AML blasts following the treatment with OTS167 as assessed by CD11b staining (P = <0.001; Figure 6C). As shown in Figure 6D, cell pellet color changed from white to red following the treatment with OTS167. Moreover, 25nM of OTS167 resulted in a significant decrease in the number of colonies grown from blasts of a patient with AML as assessed by colony forming assay (P = 0.05; Figure 6E).

### MELK inhibitor OTS167 downregulates FOXM1 activity

**Figure 7:**
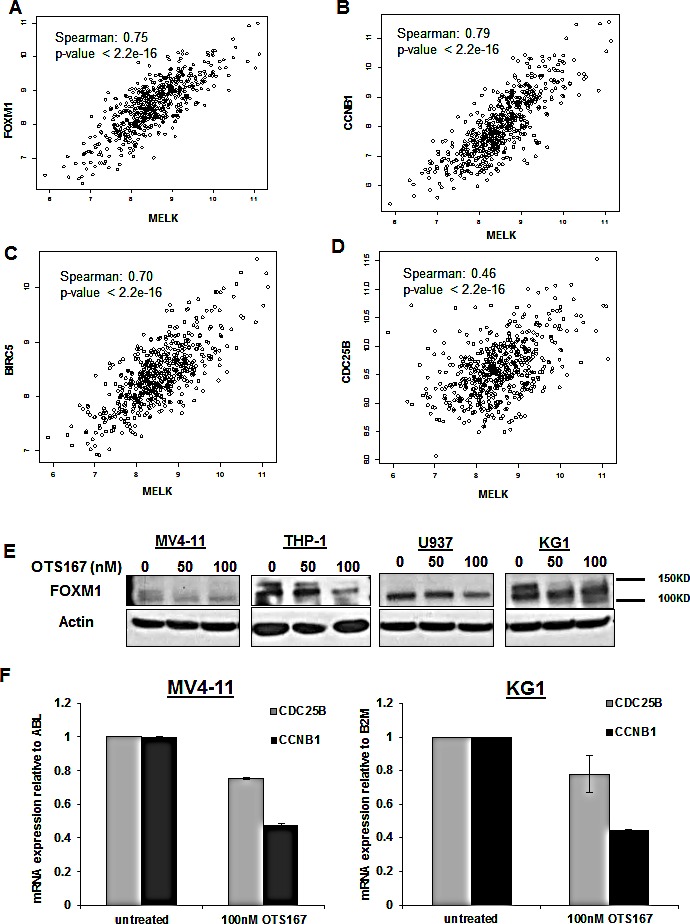
Downregulation of FOXM1 expression and activity through MELK inhibition Correlation between MELK and FOXM1 (A), CCNB1 (B), BIRC5 (C) and CDC25B (D) mRNA expression in adult AML patients. (E) FOXM1 expression measured by western blot in MV4-11, THP-1, U937 and KG1 cells treated with 50 or 100nM of OTS514 for 12 hours, (F) mRNA expression of CDC25B and CCNB1 in MV4-11 and KG1 cells treated with OTS167 measured by qRT-PCR and normalized to ABL or B2M respectively.

A previous study identified FOXM1 as a substrate of MELK protein and demonstrated that MELK overexpression resulted in activation of FOXM1 and consequent upregulation of its downstream targets (CDC25B, CCNB1 and BIRC5) [15]. Furthermore, FOXM1 and its target genes such as CCNB1 and CDC25B have been implicated in promoting cell proliferation through activating cell cycle progression in AML [16]. In order to establish the rationale for a possible involvement of MELK in the activity of FOXM1 in AML, we examined whether a correlation between MELK expression and the expression of FOXM1 or its downstream target genes in AML patients exist. In the analyzed data set of adult AML patients, we found that MELK expression was strongly correlated with that of FOXM1 (Spearman's rank correlation is 0.75), CCNB1 (Spearman's rank correlation is 0.79), and BIRC5 (Spearman's rank correlation is 0.70); weaker correlation was found with CDC25B (Spearman's rank correlation is 0.46) (Figure 7A, B, C and D).

Therefore, we hypothesized that targeting MELK in AML may result in inhibition of FOXM1 activity and subsequent downregulation of FOXM1 target genes. Indeed, MV4-11, THP-1, U937 and KG1 cells treated with 100 nM of OTS167 for 12 hours showed a decrease in phospho-FOXM1 (active form) and/or total FOXM1 (Figure 7E). Furthermore, the mRNA expression level of CCNB1 and CDC25B levels were also decreased in MV4-11 and KG1 cells following 18 and 6 hours treatment with 100 nM of OTS167 (P <0.001) (Figure 7F). Cyclin B1 protein level was also decreased in AML cells transfected with MELK-siRNA (Figure S5).

## DISCUSSION

Elevated MELK expression has been reported in a variety of solid tumors and has been associated with a poor prognosis for cancer patients. To our knowledge, MELK expression and activity have never previously been examined in hematologic malignancies. According to the TCGA database, *MELK* alteration (mutation, deletion or amplification) was observed in the range of 1-8% of cases in various types of cancer. In AML, no *MELK* mutation has been reported in the TCGA database, *but MELK* transcription was up-regulated in 4% of the cases. In this study we examined *MELK* expression in AML cells from 559 patients with different molecular and cytogenetic abnormalities. Though variable expression levels were observed in the different subsets of AML, *MELK* expression level was relatively higher in AML with a complex karyotypes, t(6,9) and del(5q)/−5, all of which are associated with chemotherapy-resistant disease and inferior clinical outcome. This observation was consistent with our finding that AML patients with higher *MELK* expression revealed shorter EFS and OS than those with lower *MELK* expression. This finding is also consistent with the negative prognostic value of elevated *MELK* expression in breast and prostate cancers, and glioblastoma [3, 8, 10, 21].

In this study, we also analyzed MELK expression at the RNA and protein levels in several AML cell lines and in primary blasts from patients with AML. Consistent with previous studies showing MELK to be expressed in the stem-cell enriched glioblastoma cells (CD133 positive cancer cells) [9], we found significantly higher MELK expression in the less differentiated leukemic cell population (enriched for CD34+ cells) than in the more differentiated cell population (CD34- cells), implying a possible role of the *MELK* gene in stem cell maintenance. Indeed, targeting MELK with a small molecule inhibitor, OTS167, resulted in a significant decrease in colony formation and migration of AML cells. Thus, we suggest that *MELK* could be a potential target for the treatment of AML.

We applied two loss-of-function approaches, one targeting *MELK* expression with siRNA and the other targeting the MELK kinase activity with a small molecule inhibitor, and examined the role of MELK in preclinical AML models. Both approaches demonstrated a significant anti-growth activity in AML cells including a dose- and time- dependent increase in cell death as well as an enhancement of myeloid differentiation in AML cell lines and primary AML blasts.

The correlation between MELK expression and FOXM1 expression in AML patients is consistent with the previously reported association between the two proteins in glioma cells [15] [22]. Although the role of MELK has not been examined in AML, its substrate FOXM1 has been shown to be upregulated in AML and possibly associated with worse clinical outcome [23, 24]. Furthermore, FOXM1 and its downstream target genes have been shown to be involved in growth and proliferation of leukemic cells [16].

In conclusion, given the high expression of MELK and the pro-apoptotic effect of its inhibition, targeting MELK may provide a new therapeutic approach. These results suggest that further clinical evaluation of the small molecule MELK inhibitor OTS167, which is currently undergoing a phase I clinical trial in solid cancer, as a novel targeted therapy for AML patients is warranted.

## MATERIALS AND METHODS

### Analysis of publically available gene expression data sets

To analyze the differential expression of *MELK* in AML subgroups, we used the publically available gene expression data set GSE37642. All patients of this cohort were treated on AMLCG protocols. The clinical and molecular characteristics of these patients were previously reported (GSE37642) [20].

### Cell Lines and Primary Blasts for *in vitro* experiments

AML cell lines (ATCC, Manassas, VA), ATCC performs cell line characterizations using short tandem repeat (STR). Cells were passaged in our laboratory for fewer than 6 months after receipt. Cells were cultured in RPMI medium supplemented with 10-20% fetal bovine serum (FBS) (**Life Technologies,** Grand Island, NY). Blast cells from AML patients were maintained in RPMI medium supplemented with 20% FBS, and 1x StemSpan CC100 (StemCell Technologies, Vancouver*,* Canada). AML blast cells used in the experiments were obtained by apheresis of blood or bone marrow samples collected from patients treated at the University of Chicago (U of C) and stored in the U of C Leukemia Tissue Bank. Informed consent to use the tissue for investigational studies was obtained from each patient according to U of C institutional guidelines.

### Reagents

siRNA sequence targeting MELK was as follows: *MELK* siRNA#1 (UGCAGCUAGAUAGGAUGUC), siRNA#2 (CCAUGUGCUAGAGACAGCCAACAAA) [25] and siRNA#3 (CUGGAUCAUGCAAGAUUACAA) [25] that were purchased from Sigma-Aldrich (St. Louis, MO). OTS167 was provided by Oncotherapy Science (Kawasaki, Japan).

### Transient Transfection, RNA Interference

Transient transfection of cells was performed utilizing 1 nmol of siRNA and 100 ul Gene Pulser buffer per reaction, and the cells were electroporated using the Bio-Rad Gene Pulse Xcell (Bio-Rad, Hercules, CA) or Amaxa Nucleofector Kit (Lonza, Basel Switzerland) according to manufacturer's instructions.

### RNA Extraction, RNA Expression Quantification

Total RNA was extracted using Trizol reagent (**Life Technologies**). *MELK* mRNA expression in AML cells was measured by ViiA 7 system according to the manufacturer's instructions. Each cDNA was synthesized using SuperScript III reagents (**Life Technologies**) according to the manufacturer's instructions. Quantitative Real-Time PCR (qRT-PCR) was performed using commercially available TaqMan Gene Expression Assay primers and probes with the ViiA 7 system (**Life Technologies**). The expression levels were normalized to *18S* rRNA, *ABL* gene or *B2M* gene.

### Western Blot Analysis and Antibodies

Western-blot analyses were performed as previously described [26]. The following antibodies were used: mouse monoclonal MELK antibody (Oncotherapy Science), and rabbit polyclonal FOXM1 antibody (Santa Cruz, Dallas, TX), mouse Cyclin B1 antibody (Santa Cruz), Caspase-3 and cleaved Caspase-3 (Cell Signaling, Danvers, MA) mouse monoclonal β-Actin (AC-15) (Sigma-Aldrich).

### Clonogenic and Viability Analyses

Methylcellulose clonogenic assays were carried out by plating 2×10^4^ primary blasts in 0.9% MethoCult (StemCell Technologies, Vancouver, Canada) [27]. Colony numbers were scored 10 days later, by counting all colonies per well. For viability analysis, MTT assay was performed in a 96-well plate and 5×10^4^ cells were plated per well. Cell counting kit-8 (Dojindo Molecular Technologies, Inc., Kumamoto, Japan) was used for MTT reaction.

For viability and apoptosis analyses, cells were collected, spun down then washed with PBS and resuspended in 50 μl binding buffer containing 2 μL of Annexin V (eBioscience, San Diego, CA), and 5 μL propidium iodide (PI) (eBioscience). After 20 min incubation, fluorescence was quantified by flow cytometry on a FACSCalibur instrument [26].

### Migration assay

Migration assays were performed in transwell plates (Costar, Cambridge, MA) of 6.5-mm diameter with 5-μm pore filters as previously described [28]. Approximately 100,000 AML cells in 0.1 mL RPMI with 10% FBS were added to the upper compartment, and 0.6 mL of the same medium including SDF-1a (400nM final concentration) was added to the lower compartment. SDF-1α was purchased from PeproTech (Rocky Hill, NJ). Transwell plates were incubated at 37°C in the condition of 5% CO_2_ for 4 hours. Cells in the bottom well (migrated cells) were then imaged under the microscope.

### Immunofluorescent Staining, Flow Cytometry

Cells were washed with PBS, spun down and stained with CD11b antibodies (eBioscience, San Diego, CA) with 20-min incubation at room temperature; cells were then washed with PBS and resuspended in PBS. Fluorescence was quantified by flow cytometry on a FACSCalibur instrument [29].

### Cell Cycle Analysis

Cells were treated with 50nM of OTS167, 24 hours later cells were incubated with Brdu for 45 minutes, then collected, washed with PBS, fixed and stained according to BD Pharmingen FITC Brdu Flow kit instruction (BD, San Jose, CA). Samples were then analyzed by flow cytometry on a FACSCalibur instrument

### Statistical Analysis

Definitions of clinical end points (event free survival (EFS) and overall survival (OS)) are as previously reported [30]. Wilcoxon test was used to compare *MELK* expression of each cytogenetically abnormal subgroup to patients with cytogenetically normal (CN-) AML (reference). The differences in baseline clinical and molecular features between the *MELK* higher and lower groups were tested using the Fisher's exact and Wilcoxon rank sum tests for categorical and continuous variables, respectively. Estimated probabilities of EFS and OS were calculated using the Kaplan-Meier method, and the log-rank test was used for evaluation of differences between survival distributions. Mechanistic and biological experiments were analyzed with paired and unpaired two-sided t-tests. *P* values < .05 were considered statistically significant. Experiments were performed in triplicate (except for when patient blasts were used, these experiment were done in duplicate when possible), results were presented by Mean ± SE.

## SUPPLEMENTARY MATERIAL FIGURES


