# Supplementary material for: Preclinical efficacy of maternal embryonic leucine-zipper kinase (MELK) inhibition in acute myeloid leukemia
Source: Oncotarget. 2014 Oct 28;5(23):12371–82. doi: 10.18632/oncotarget.2642 (PMC4323011; doi:10.18632/oncotarget.2642)
Supplement: Supplementary file 1 [file oncotarget-05-12371-s001.pdf]

Preclinical efficacy of maternal embryonic leucine-zipper kinase (MELK) inhibition in acute myeloid leukemia

Supplementary Material

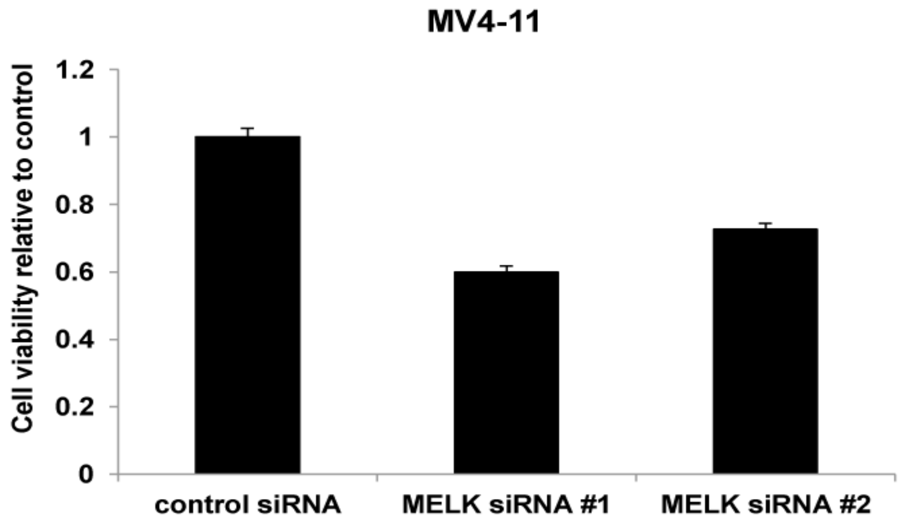

Figure S1: MELK knock-down decreased cell viability. MV4-11 cell transfected with two different siRNAs against MELK and cell viability was assessed by MTT assay 48 hours later.

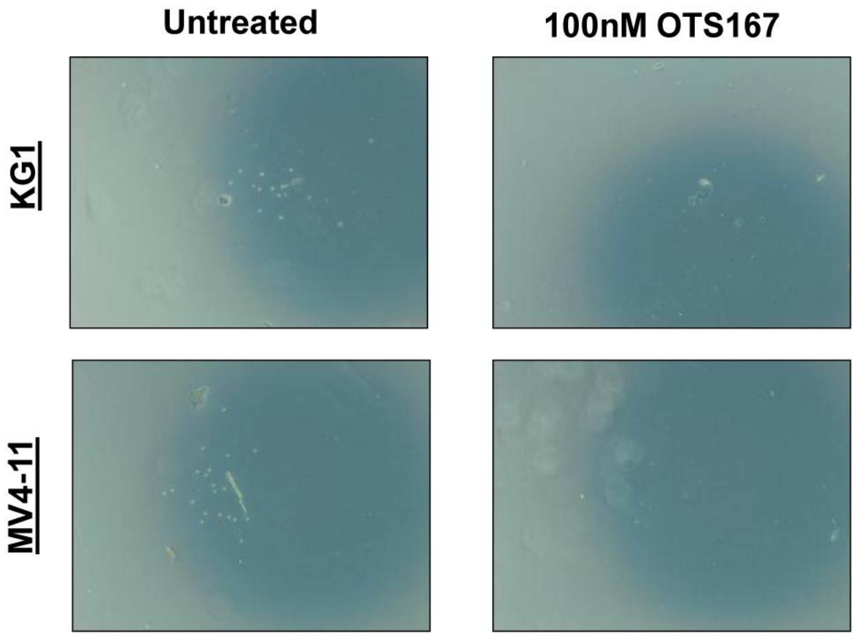

Figure S2: MELK inhibitor OTS167 reduces cell migration. KG1 and MV4-11 cells were treated with 100nM of OTS167 for 6 hours, then cell migration was assessed in trans-well plate, image shows cells in the bottom well.

(12 hours)

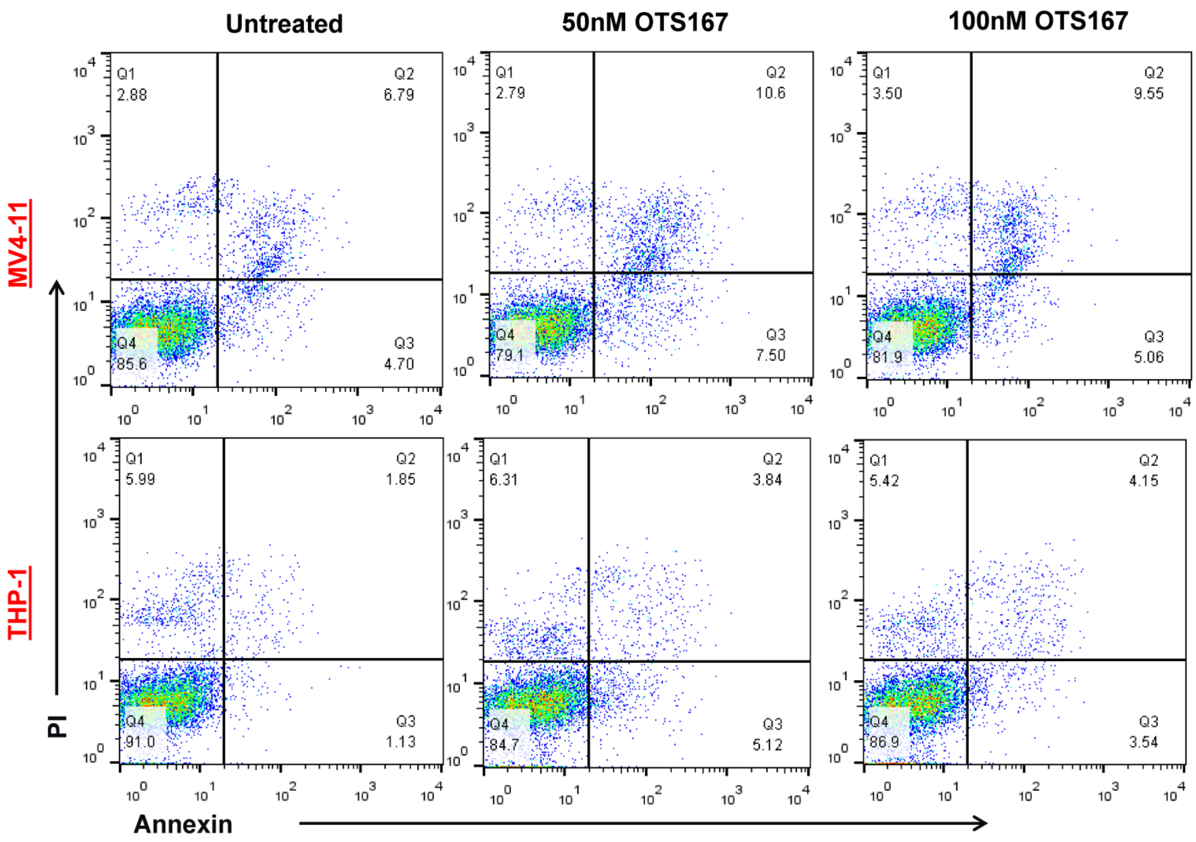

Figure S3: The effect of MELK inhibitor OTS167 on cell apoptosis 12 hours following treatment. MV4-11 and THP-1 cells were treated with 50 and 100nM of OTS167 for 12 hours, then apoptosis was assessed by Annexin and PI staining.

24 hours

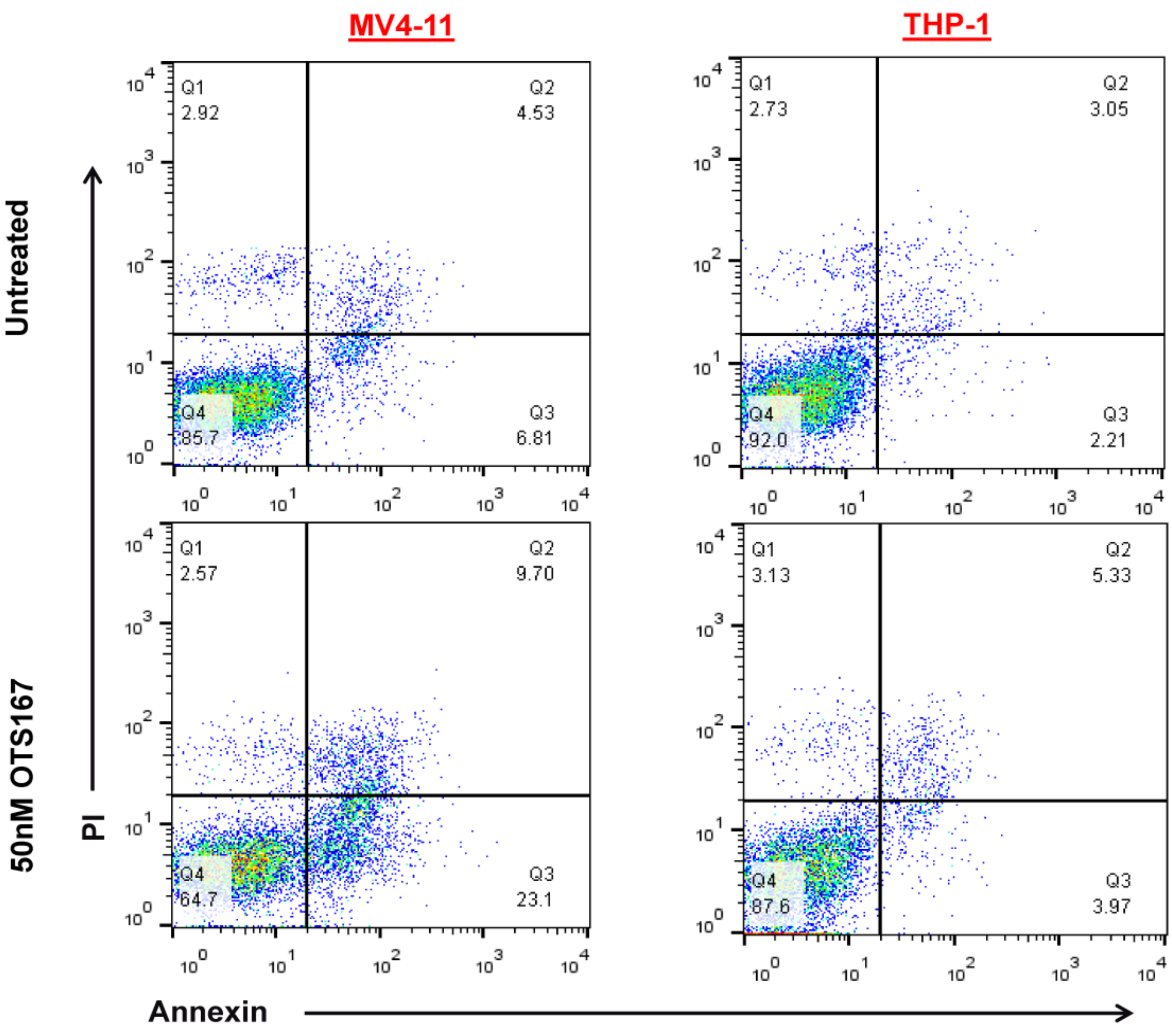

Figure S4: The effect of MELK inhibitor OTS167 on cell apoptosis 24 hours following treatment. MV4-11 and THP-1 cells were treated with 50nM OTS167 for 24 hours, then apoptosis was assessed by Annexin and PI staining.

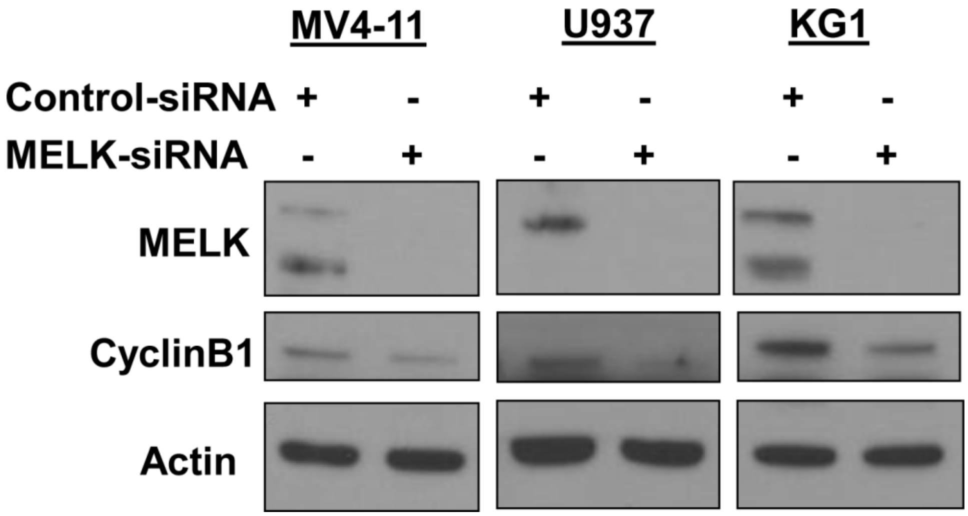

Figure S5: The effect of MELK inhibition on CyclinB1 expression. MV4-11, U937 and KG1 cells were transfected with MELK-siRNA, and cyclinB1 expression was assessed 48 hours following transfection.
